# Engineered CD147-CAR macrophages for enhanced phagocytosis of cancers

**DOI:** 10.1007/s00262-024-03759-6

**Published:** 2024-07-02

**Authors:** Koollawat Chupradit, Saitong Muneekaew, Methichit Wattanapanitch

**Affiliations:** Siriraj Center for Regenerative Medicine, Research Department, Faculty of Medicine Siriraj Hospital, Mahidol University, Bangkok, Thailand

**Keywords:** Chimeric antigen receptor, CAR-macrophages, CD147, Phagocytosis, Cancer, Immunotherapy

## Abstract

Chimeric antigen receptor (CAR) T cell therapy has shown promising results in hematologic malignancies, but its effectiveness in solid cancers remains challenging. Macrophages are immune cells residing within the tumor microenvironment. They can phagocytose tumor cells. Recently, CAR macrophages (CAR-M) have been a promising candidate for treating solid cancers. One of the common cancer antigens overexpressed in various types of cancer is CD147. CAR-T and NK cells targeting CD147 antigen have shown significant efficacy against hepatocellular carcinoma. Nevertheless, CAR-M targeting the CD147 molecule has not been investigated. In this study, we generated CAR targeting the CD147 molecule using the THP-1 monocytic cell line (CD147 CAR-M). The CD147 CAR-M exhibited typical macrophage characteristics, including phagocytosis of zymosan bioparticles and polarization ability toward M1 and M2 phenotypes. Furthermore, the CD147 CAR-M demonstrated enhanced anti-tumor activity against K562 and MDA-MB-231 cells without exhibiting off-target cytotoxicity against normal cells. Our research provides valuable insights into the potential of CD147 CAR-M as a promising platform for cancer immunotherapy, with applications in both hematologic malignancies and solid cancers.

**Graphical abstract:**

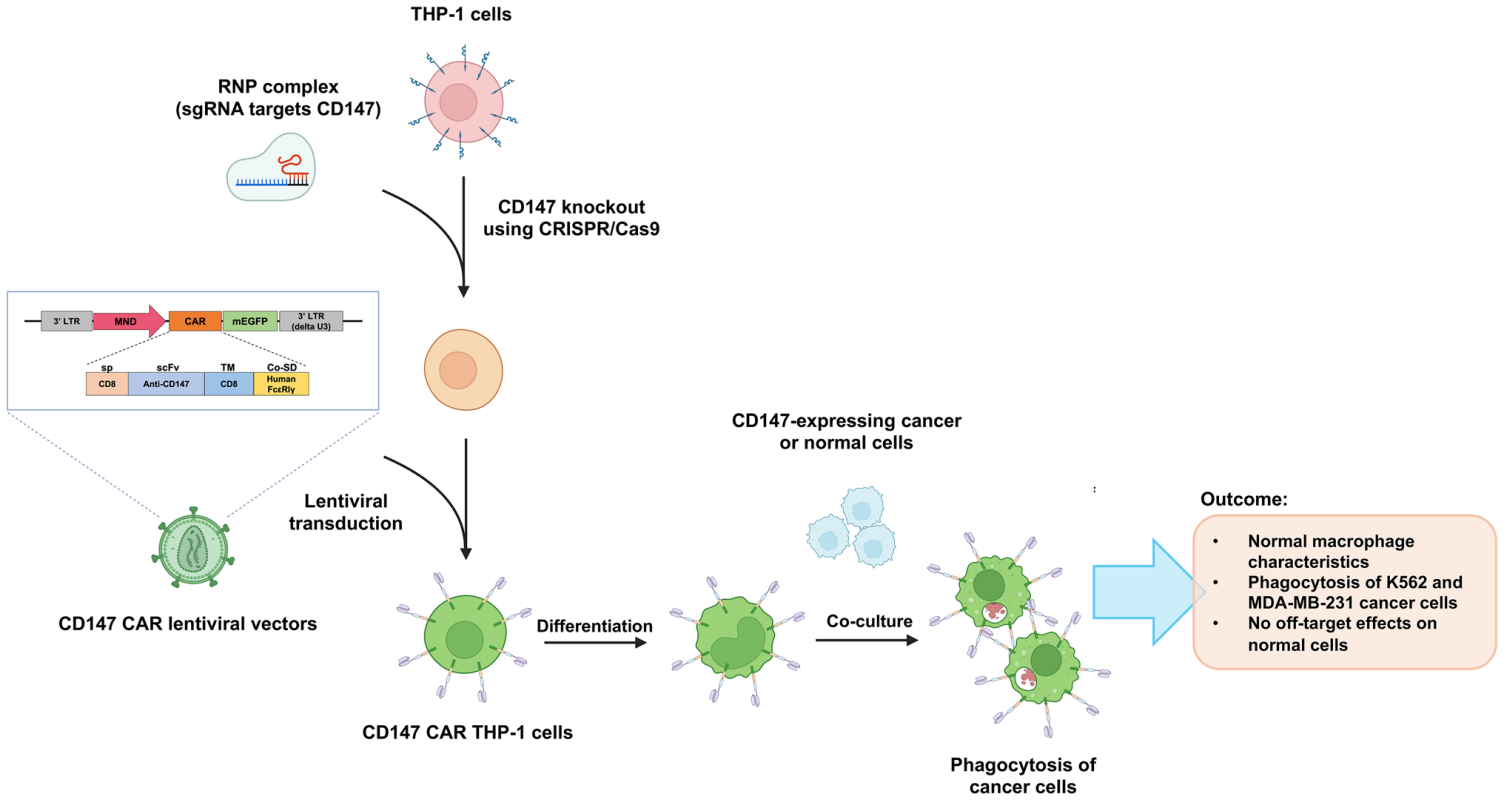

**Supplementary Information:**

The online version contains supplementary material available at 10.1007/s00262-024-03759-6.

## Introduction

Immunotherapy is a cornerstone of modern cancer treatment, with chimeric antigen receptor (CAR) technology showing promising anti-tumor effects among recent approaches. CAR is an engineered receptor that recognizes the target antigen, enabling cytotoxicity against the target cells. Recently, six CAR-T cell therapies have been approved by the US Food and Drug Administration (FDA) for treating B-cell malignancies [1–4]. Nevertheless, CAR-T cell therapy has several limitations, including antigen escape, on-target off-tumor cytotoxicity, poor trafficking, and tumor infiltration [5, 6]. Moreover, CAR-T cell therapy is less effective in treating solid tumors than hematologic malignancies [7–9]. Recently, CAR macrophages (CAR-M) has been developed to treat solid cancers [10, 11]. CAR-M could penetrate the solid tumor, phagocytose it, and present antigens to T cells, thus enhancing tumor killing [12, 13]. Previous studies demonstrated that the anti-CD19 and anti-CD22 CAR-M exhibited increased phagocytosis of antigen-coated beads and tumor cells compared to the wild-type macrophages [14]. The first clinical trial for the anti-HER2 CAR-M in patients with solid tumors has been initiated [15]. However, engineering peripheral blood monocyte-derived macrophages (MDMs) is challenging due to their resistance to transduction [16]. In addition, MDMs have limited proliferation, posing challenges for clonal isolation and expansion. In a recent development, Zhang and colleagues created CAR-iPSCs and differentiated them into macrophages, the so-called CAR-iMACs. These cells were capable of differentiating into the M1 macrophage subtype and phagocytosing the CD19-expressing solid tumors [17]. These studies indicate that CAR-M can promote phagocytic activity in an antigen-specific manner.

Identifying tumor target antigens is pivotal for understanding cancer biology and developing therapeutics. CD147, also known as Basigin, is a transmembrane glycoprotein that plays multifunctional roles in immune cell biology [18]. CD147 is expressed in various cell types, such as epithelial and endothelial cells [19]. Overexpression of CD147 is observed in several cancers and is associated with a poor prognosis [20, 21]. During the past decades, there has been extensive research into the roles of CD147, particularly in relation to the proliferation, invasiveness, and metastasis of cancer cells [22, 23]. CD147 on cancer cells plays a crucial role in activating the matrix metalloproteinases, leading to tumor invasion, growth, and metastasis [24–26]. Thus, CD147 represents a promising therapeutic target in cancer therapy. The previous study demonstrated that an anti-CD147 monoclonal antibody could target CD147 molecules expressed on hepatocellular carcinoma (HCC) and decrease tumor metastasis in a rabbit xenograft model [27]. Other studies employed anti-CD147 antibody (metuzumab) to inhibit the growth of solid tumors, A549 and NCI-H520, in a mouse xenograft model [28]. More recently, CAR-T and CAR-natural killer (NK) cells targeting CD147 eliminated HCC cell lines in vitro and HCC tumors in xenograft mouse models [29]. In addition, the intrabody containing the CD147 single-chain variable fragment (scFv) could decrease the expression of extracellular matrix metalloproteinase inducer (EMMPRIN) on the cell surface, leading to reduced cell proliferation, invasion, and metastasis, as well as induced apoptosis in the colorectal adenocarcinoma cell line Caco-2 [30, 31]. These findings indicate the therapeutic potential of CARs targeting CD147. However, the effectiveness of CAR-M targeting the CD147 molecule has not been investigated.

To address the limitations of CAR-T cells, our study aimed to explore the potential of using CD147 CAR in the THP-1 monocytic cell line as a proof-of-concept study for anti-cancer therapy using CAR-M. We engineered THP-1 cells to express CD147 CAR and assessed their functions, including phagocytosis of zymosan bioparticles, differentiation into M0, and polarization towards M1 and M2 macrophage subtypes. Additionally, we evaluated the specificity of the CD147 CAR THP-1 macrophages by assessing their ability to phagocytose cancer cells that overexpress CD147. Our findings offer valuable insights into the mechanism and specificity of CD147 CAR therapy for cancer, serving as a crucial platform for adoptive transfer-based treatment of solid cancers.

## Methods

### Cell culture

Human peripheral blood mononuclear cells (PBMCs) were obtained from healthy donors following the protocol approved by the Siriraj Institutional Review Board (SiRB), no. Si 634/2022, in accordance with the Helsinki Declaration of 1975. All participants were given a participant information sheet, provided with explanations, and signed the informed consent. Human THP-1 cells (ATCC TIB-202), K562 (ATCC CCL-243), and human PBMCs were cultured in a complete RPMI-1640 medium (Gibco, 1TFS-1CC-31800022) containing RPMI-1640 basal medium, 10% fetal bovine serum (FBS) (Gibco, F7524-500ML), 2 mM GlutaMax (Gibco, 1IVG7-35050-061), and 100 units/mL of penicillin-streptomycin (Gibco, 1IVG7-15140-122). Human embryonic kidney (HEK293T) (ATCC CRL-3216), MCF-7 (ATCC HTB-22), and MDA-MB-231 (ATCC HTB-26) were cultured in a complete Dulbecco’s modified Eagle’s medium (DMEM) (Gibco, 1TFS-1CC-12500062) containing DMEM basal medium supplemented with 10% FBS, 2 mM GlutaMax, and 100 units/mL of penicillin-streptomycin. MCF10A cells (ATCC CRL-10317) were cultured in a complete DMEM/Ham’s F-12 medium (Gibco, 11330-032) containing DMEM/Ham’s F-12 basal medium supplemented with 100 ng/mL cholera toxin (Sigma, C-8052), 20 ng/mL epidermal growth factor (EGF) (Peprotech, AF-100-15), 0.01 mg/mL insulin (Sigma, I-1882), 500 ng/mL hydrocortisone (Sigma, H-0888), and 5% horse serum (Invitrogen, 16050-122). Experiments were conducted using MCF10A cells within eight passages of culture. All cells were maintained in a 37 °C humidified incubator with 5% CO_2_.

### Generation of CD147 knockout (KO) THP-1 cells using CRISPR/Cas9 technique

**Fig. 1:**
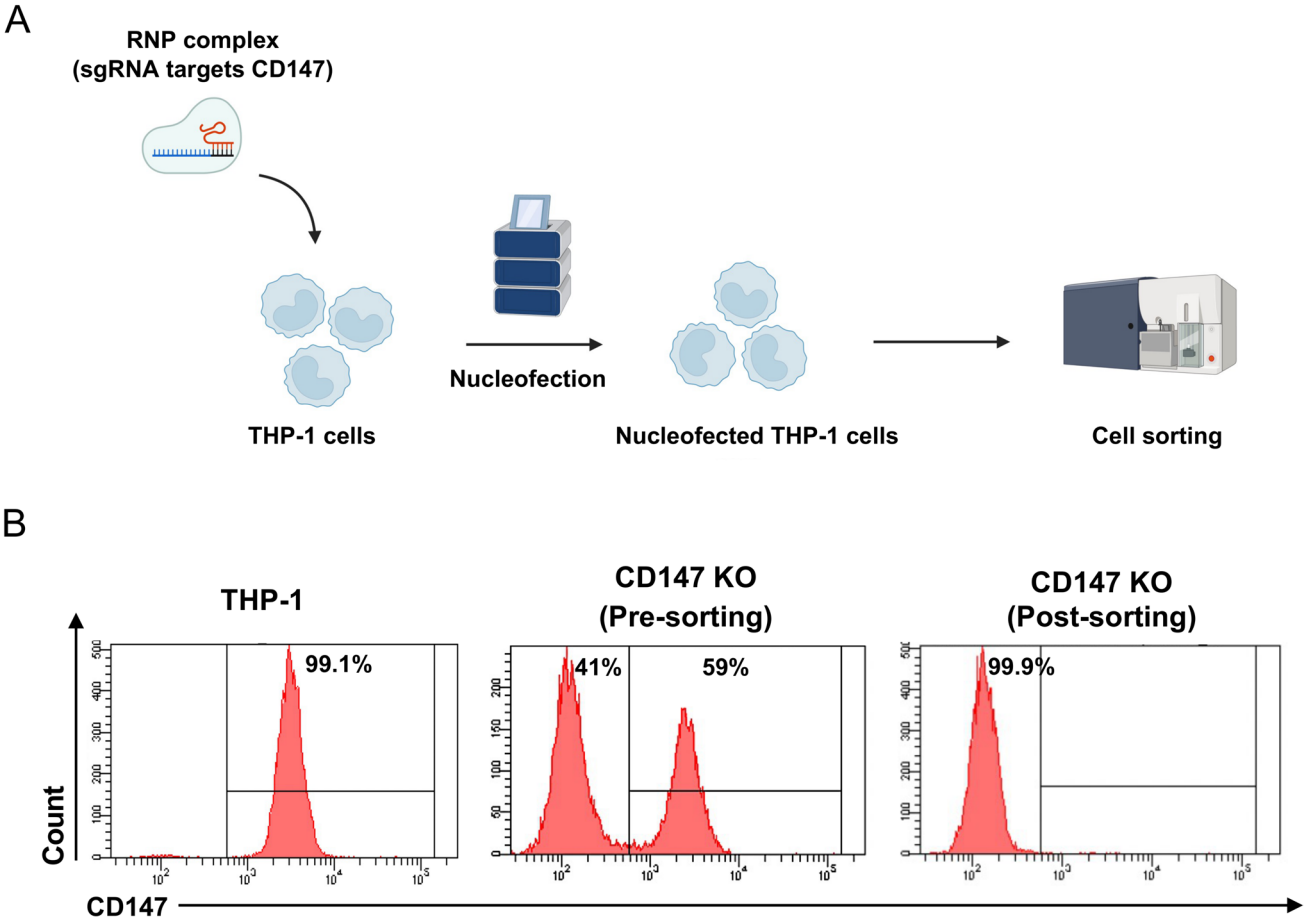
CRISPR/Cas9-mediated CD147 knockout in the THP-1 cell line. **A** Schematic diagram of CD147 knockout in the THP-1 cell line using nucleofection (Created by BioRender.com/Mahidol University). **B** Flow cytometric analysis of CD147 expression in the THP-1 cells before nucleofection, after nucleofection (pre-sorting) and post-sorting

The single guide RNA (sgRNA) targeting CD147 was synthesized using the GeneArt Precision gRNA Synthesis kit (Invitrogen, A29377). The sgRNA target sequence was 5′-GCGAGGAATAGGAATCATGG-3′, which targets the exon 1 of the N-terminus of the CD147 molecule [32]. We performed CRISPR/Cas9-mediated gene knockout according to the previous study [33]. Briefly, the ribonucleoprotein (RNP) complex was prepared by mixing 150 µg/mL of the SpCas9 protein (IDT, IDE1081058) with 90 µg/mL of the sgRNA. The mixture was incubated for 10 min at room temperature. Subsequently, 10^6^ THP-1 cells were nucleofected with the RNP complex using the P3 Primary Cell 4D-Nucleofector™ X Kit S (Lonza, V4XP-3032) and the FF100 program of the 4D-Nucleofector™ X Unit (Fig. 1A). On day 11 post-nucleofection, the nucleofected cells were stained with human anti-CD147 Alexa Flour 488-conjugated antibody (R&D systems, FAB3195G). The CD147 expression was determined by flow cytometry (BD Biosciences, LSRFortessa). The CD147 KO THP-1 cells were sorted for further experiments using a cell sorter (BD Biosciences, FACSAria III).

### Preparation of CD147 CAR lentiviral vectors

The CGW CD147 CAR transgene vector consists of the murine myeloproliferative sarcoma virus (MND) promoter, CD8 signal peptide (sp), the scFv targeting CD147 (M6-1B9) [34], CD8 transmembrane (CD8 TM), human Fc-gamma receptors (FcεRIγ) intracellular signaling domain, and monomeric enhanced green fluorescent protein (mEGFP) [14]. For lentiviral production, HEK293T cells were seeded onto two 10-cm dishes at a density of 3.5 × 10^6^ cells per dish. The next day, we co-transfected four plasmid vectors, including the CGW CD147 CAR transgene vector (11.3 µg/dish), the packaging construct pMDLg/pRRE (3.65 µg/dish), pRSV-Rev (3.65 µg/dish), and pMD.2G (3.9 µg/dish) into the HEK293T cells using Lipofectamine 3000 transfection reagent (Invitrogen, L3000015). Two days post-transfection, the culture supernatant containing CD147 CAR lentiviral vectors was collected for further experiment.

### Generation of the CD147 CAR THP-1 cells

We transduced 5 × 10^5^ CD147 KO THP-1 cells with the CD147 CAR lentiviral vectors obtained from the culture supernatant. The transduction was performed in a 24-well plate with a 1:2 dilution in a final volume of 500 µL of a complete RPMI-1640 medium containing 8 µg/mL of polybrene (Sigma-Aldrich, H9268). The cells were incubated at 37 °C and 5% CO_2_ for 24 h. After incubation, the transduced cells were washed three times with RPMI-1640 basal medium and cultured in a complete RPMI-1640 medium. The transduction efficiency was determined three days post-transduction by observing the mEGFP-positive cells using fluorescence microscopy and flow cytometry. The mEGFP-positive cells were sorted using a cell sorter.

### Binding assay of CD147 CAR and CD147 molecule

THP-1 cells were seeded onto a Poly-L-lysine (50 µg/mL, Sigma-Aldrich, P8920)-coated glass coverslip at a density of 5 × 10^5^ cells/well of a 24-well plate in a serum-free RPMI medium for 24 h. The cells were fixed with 4% paraformaldehyde/PBS for 15 min at room temperature. The coverslip was gently washed twice with sterile PBS. Next, 10 µg/mL recombinant CD147 extracellular domain-human IgG Fc fusion protein (CD147Rg) was added and incubated for 1 h. After three washes with sterile PBS, the pre-adsorbed Rabbit Anti-human IgG H&L (Dylight (DL) 650) (1: 500 dilution) (Thermo Fisher Scientific, SA5-10113) and Hoechst33342 (1: 1000 dilution) (Invitrogen, 1IVM-R37605) were added and further incubated for one hour. The coverslip was washed three times with PBS and placed upside down on the glass slide with 4 µL of 50% glycerol/PBS. The fluorescent images were acquired on a Zeiss LSM 800 confocal microscope.

### Phagocytosis of the pHrodo Red zymosan bioparticles

For differentiation into macrophages, we cultured 5 × 10^5^ THP-1 cells/well of a 24-well plate containing 400 µL of complete RPMI-1640 medium and 100 ng/mL phorbol 12-myristate 13-acetate (PMA) (BioGems, 1652981). On day 2, the cells were washed once with 500 µL complete medium and incubated in 225 µL of complete RPMI-1640 medium containing 25 µL of pHrodo™ Red zymosan A Bioparticles™ conjugate for Phagocytosis (Invitrogen, P35364) for 3 h. Afterward, the excess bioparticles were washed once with the medium. The red fluorescent signal was observed using a fluorescence microscope. The cells were also harvested using 2 mM EDTA in PBS for flow cytometric analysis.

### Polarization of THP-1 cells into M1 and M2 macrophages

For macrophage differentiation, 1.3 × 10^6^ THP-1 cells were cultured in a well of 6-well plate containing 2 mL of complete RPMI-1640 medium supplemented with 10 ng/mL PMA for 24 h. For M1 polarization, the THP-1-derived macrophages were cultured in a complete RPMI-1640 medium supplemented with 20 ng/mL interferon-gamma (IFN-γ) (Biolegend, 570206) and 15 ng/mL lipopolysaccharide (LPS) (Sigma, L2630-10MG). For M2 polarization, the THP-1-derived macrophages were cultured in a complete RPMI-1640 medium supplemented with 25 ng/mL IL-4 (Biolegend, 574008) and 25 ng/mL IL-13 (Biolegend, 571102). The cells were further cultured for 72 h. Afterward, the cells were harvested for flow cytometric analysis of macrophage markers using PerCP anti-human CD45 (BIOL-368506), APC/Cyanine7 (Cy7) anti-human CD11b (BIOL-101225), PE/Cy7 anti-human CD80 (BIOL-375407), Brilliant Violet (BV) 650™ anti-human HLA-DR (BIOL-307649), APC anti-human CD163 (BIOL-333609), and BV 510™ anti-human CD206 (BIOL-321137) (all from Biolegend).

### Phagocytosis of cancer and normal cell lines by THP-1-derived macrophages

THP-1 cells were stained with 2.5 µM Carboxyfluorescein succinimidyl ester (CFSE) (Biolegend, BIOL-423801) for 20 min and cultured in a complete RPMI-1640 medium overnight. The following day, the cells were seeded at a density of 1 × 10^4^ cells/well of a 24-well plate containing 100 µL of complete RPMI-1640 medium supplemented with 10 ng/mL PMA. The next day, the medium was replaced with 200 µL of complete RPMI-1640 medium. The cancer cell lines, including MCF-7, MDA-MB-231, and K562, or normal cells, including MCF10A and PBMCs, were stained with 0.5 µM of pHrodo™ iFL Red STP ester, amine-reactive dye (Invitrogen, P36011) before co-culturing with macrophages at the effector to target (E:T) ratios of 1:2 to 1:3. After 24 h of co-culture, the green and red fluorescent signals were observed using an EVOS M7000 imaging system (Thermo Fisher Scientific). We captured the images at 1-h intervals for the next 24 h and quantified the red area using the Celleste 6 image analysis software. For CD147 and CD47 surface expression, cancer cells were stained with anti-CD147 Alexa Fluor 488-conjugated and APC anti-human CD47 antibodies (Biolegend, BIOL-323123) for 15 min at 4 °C in 3% FBS/PBS and further analyzed by flow cytometry.

### Cytokine secretion by ELISA

The culture supernatant from the co-culture condition was collected and centrifuged for 5 min at 500×*g* to remove the cells and cell debris. The cytokine was measured using the DuoSet ELISA Development Kit for human IL-6 (R&D Systems, DY206-05) and TNF-⍺ (R&D Systems, DY210-05).

### Statistics

Statistical analyses were performed using the GraphPad Prism software. Statistical significance was determined by the multiple unpaired *t* test analysis. The significance levels are as follows: ^ns^indicates *P* > 0.05, *indicates *P* < 0.05, **indicates *P* < 0.01, ***indicates *P* < 0.001.

## Results

### CRISPR/Cas9-mediated CD147 knockout in the THP-1 cell line

In this study, we used a human monocytic cell line, THP-1, isolated from an acute monocytic leukemia patient, as a model to generate CD147 CAR-M and validated the phagocytic efficiency in comparison to the WT cells. Before transducing with CD147 CAR, we assessed the expression levels of CD147 molecules on the surface of THP-1 cells and found that almost 100% of the cells expressed CD147 molecules (Fig. 1B). To prevent self-phagocytosis, we used the CRISPR/Cas9 gene-editing system to knock out the CD147 gene in the THP-1 cells. On day 11 post-nucleofection, approximately 59% of the nucleofected THP-1 cells expressed CD147. The CD147-negative population was sorted for expansion, resulting in a homogeneous CD147-negative pool, as shown by flow cytometric analysis (Fig. 1B). These data suggested that the sgRNA targeting the N-terminus of the CD147 molecule resulted in a complete loss of CD147 molecules in the THP-1 cells.

### Generation of the CD147 CAR THP-1 cells

**Fig. 2:**
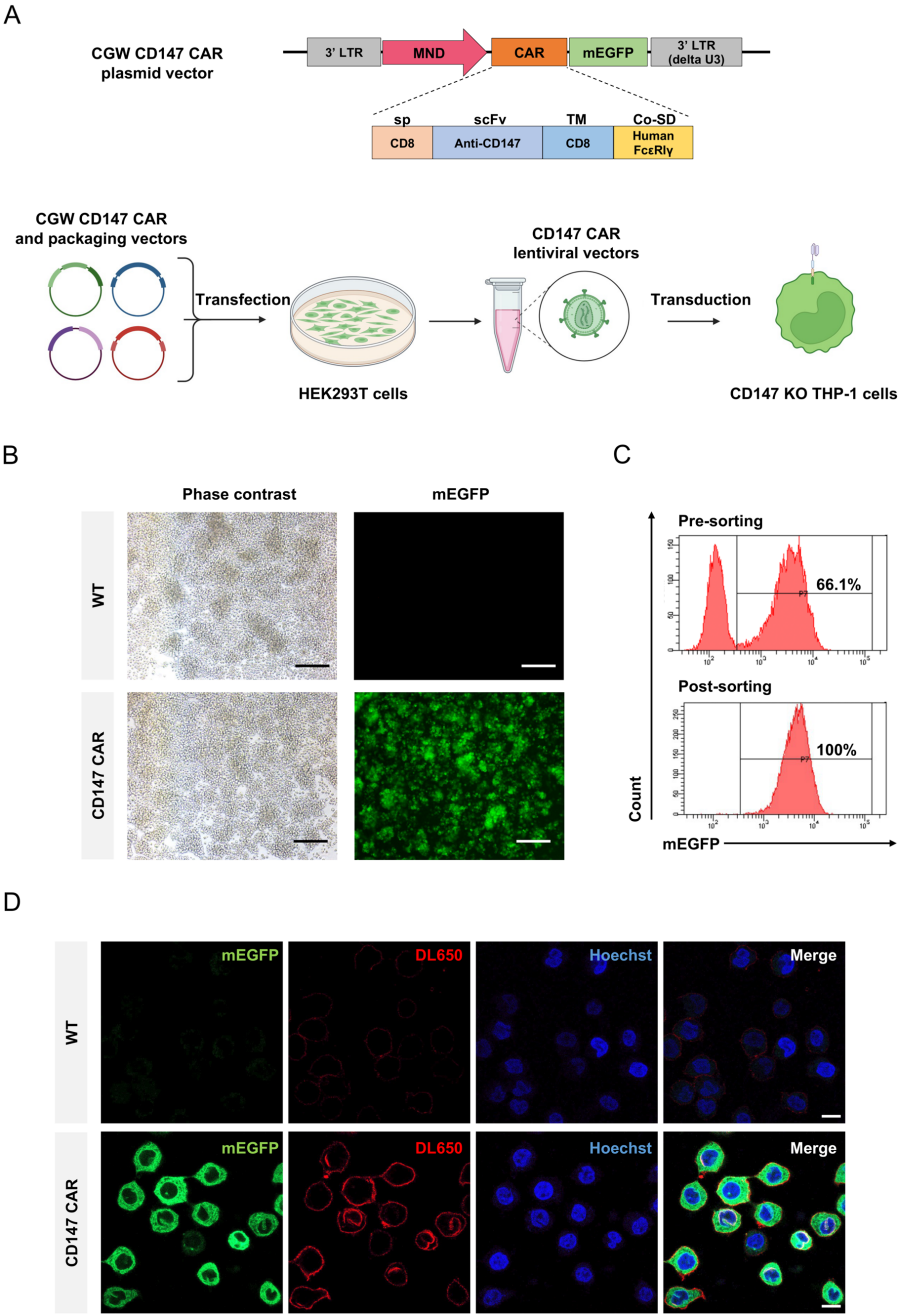
Generation of the CD147 CAR lentiviral vectors and lentiviral transduction of CD147 CAR into the CD147 KO THP-1 cells. **A** Schematic representation of the CGW CD147 CAR plasmid vector and lentiviral vector production. LTR, Long terminal repeat; MND, a modified enhancer/promoter of the murine myeloproliferative sarcoma virus promoter; CAR, chimeric antigen receptor; sp, signal peptide; scFv, single chain fragment variable; TM, transmembrane domain; Co-SD, co-stimulatory signaling domain; mEGFP, monomeric enhanced green fluorescent protein (Created by BioRender.com/Mahidol University). **B** Representative fluorescent microscopic images of the WT THP-1 and CD147 CAR-transduced THP-1 cells on day 2 post-transduction, scale bar = 200 µm. **C** Flow cytometric analysis of the CD147 CAR-transduced THP-1 cells on day 5 pre- and post-sorting. **D** Representivative confocal microscopy images demonstrating the binding of the CD147 scFv molecules to the recombinant CD147Rg in the WT THP-1 cells and the CD147 CAR THP-1 cells. (63 × oil immersion objective of the Zeiss LSM 800 confocal microscope, scale bar = 10 µm)

We first generated the CD147 CAR lentiviral vectors containing CD8 sp, scFv targeting CD147, CD8 TM domain, and human FcεRIγ intracellular signaling domain fused with the mEGFP reporter protein. This gene cassette was controlled by the MND promoter (Fig. 2A). The lentiviral vectors were transduced into the CD147 KO THP-1 cells. On day 2 post-transduction, we observed bright green fluorescent protein from the mEGFP in the transduced THP-1 cells (Fig. 2B), accounting for 66.1% of the whole population. We sorted the mEGFP-positive THP-1 cells for CD147 CAR expression (Fig. 2C) and assessed their ability to bind to the recombinant CD147Rg. The results confirmed that the CD147 CAR THP-1 cells displayed red fluorescence from DL650, indicating the specific binding activity of CD147 CAR to the recombinant CD147 Rg (Fig. 2D).

### The CD147 CAR-M exhibited normal phagocytosis towards zymosan bioparticles

**Fig. 3:**
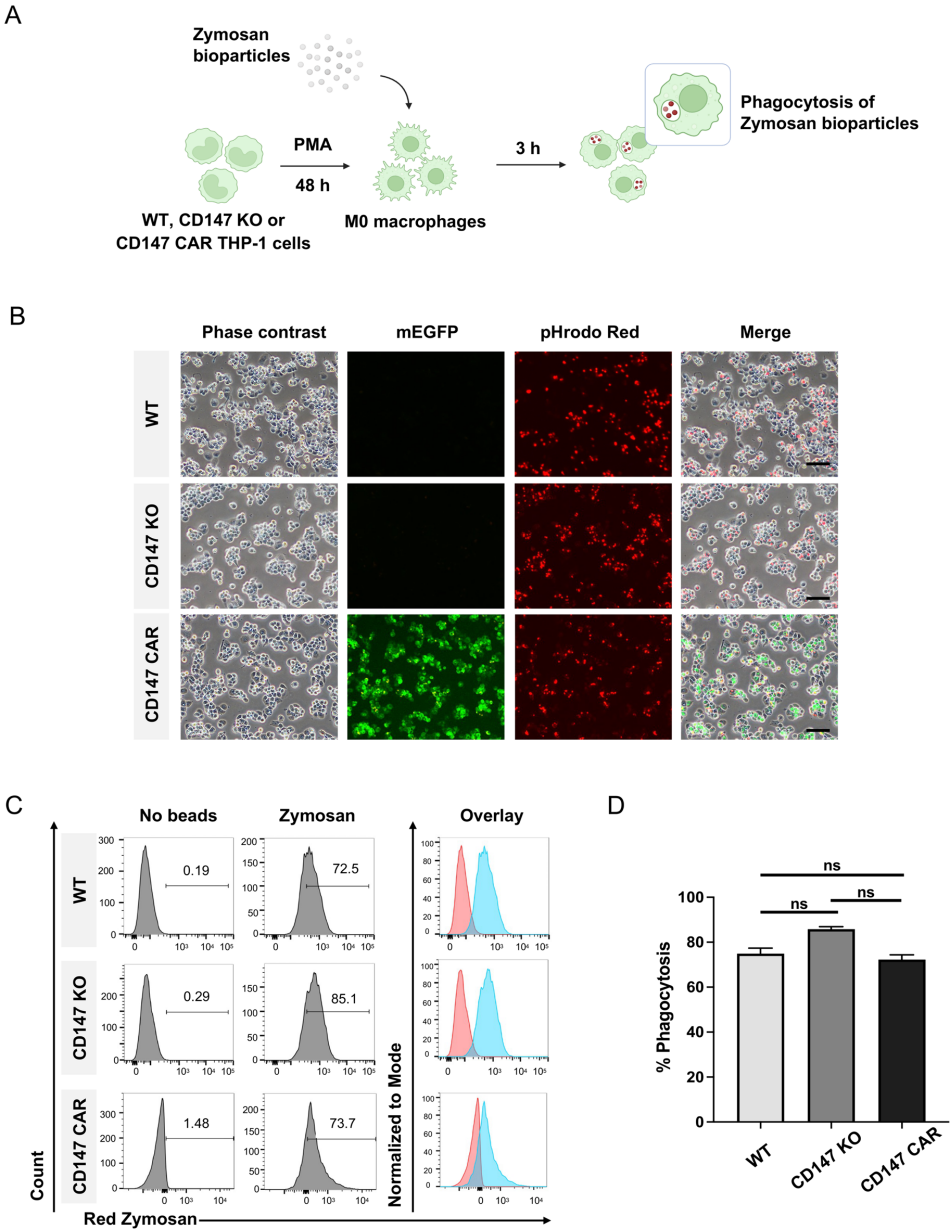
Phagocytosis of zymosan bioparticles. **A** Schematic diagram illustrating the phagocytosis process of zymosan bioparticles by the wild-type (WT), CD147 KO, and CD147 CAR-macrophages (Created by BioRender.com/Mahidol University). **B** Representative fluorescent microscopic images depicting the WT, CD147 KO, CD147 CAR macrophages co-cultured with the zymosan bioparticles, scale bar = 100 µm. **C** Representative histograms and **D** Bar graph showing phagocytic activity against zymosan bioparticles as determined by flow cytometry. Statistical significance was calculated using multiple unpaired *t* test analyses, ^ns^indicates *P* > 0.05. Data are results from three independent experiments

To determine the typical functions of the WT, CD147 KO, and CD147 CAR THP-1 cells, we induced macrophage differentiation in these cells and evaluated their phagocytic activity using zymosan-coupled bioparticles (Fig. 3A). The pHrodo fluorogenic dye, which fluoresces in response to increased acidity within the surrounding environment, was used to track the phagocytic function. After 3 h of incubation with zymosan-coupled bioparticles, the WT, CD147 KO, and CD147 CAR THP-1-derived macrophages exhibited red fluorescence from pHrodo red under a fluorescent microscope (Fig. 3B). Flow cytometric analysis revealed similar percentages of cells engulfing the bioparticles (74.93 ± 2.45%, 85.8 ± 1.13%, 72.27 ± 2.14%, respectively) (Fig. 3C, D). The results indicated that all three cell types displayed comparable phagocytic function towards zymosan bioparticles.

### The CD147 CAR THP-1 cells exhibited typical macrophage characteristics after polarization

**Fig. 4:**
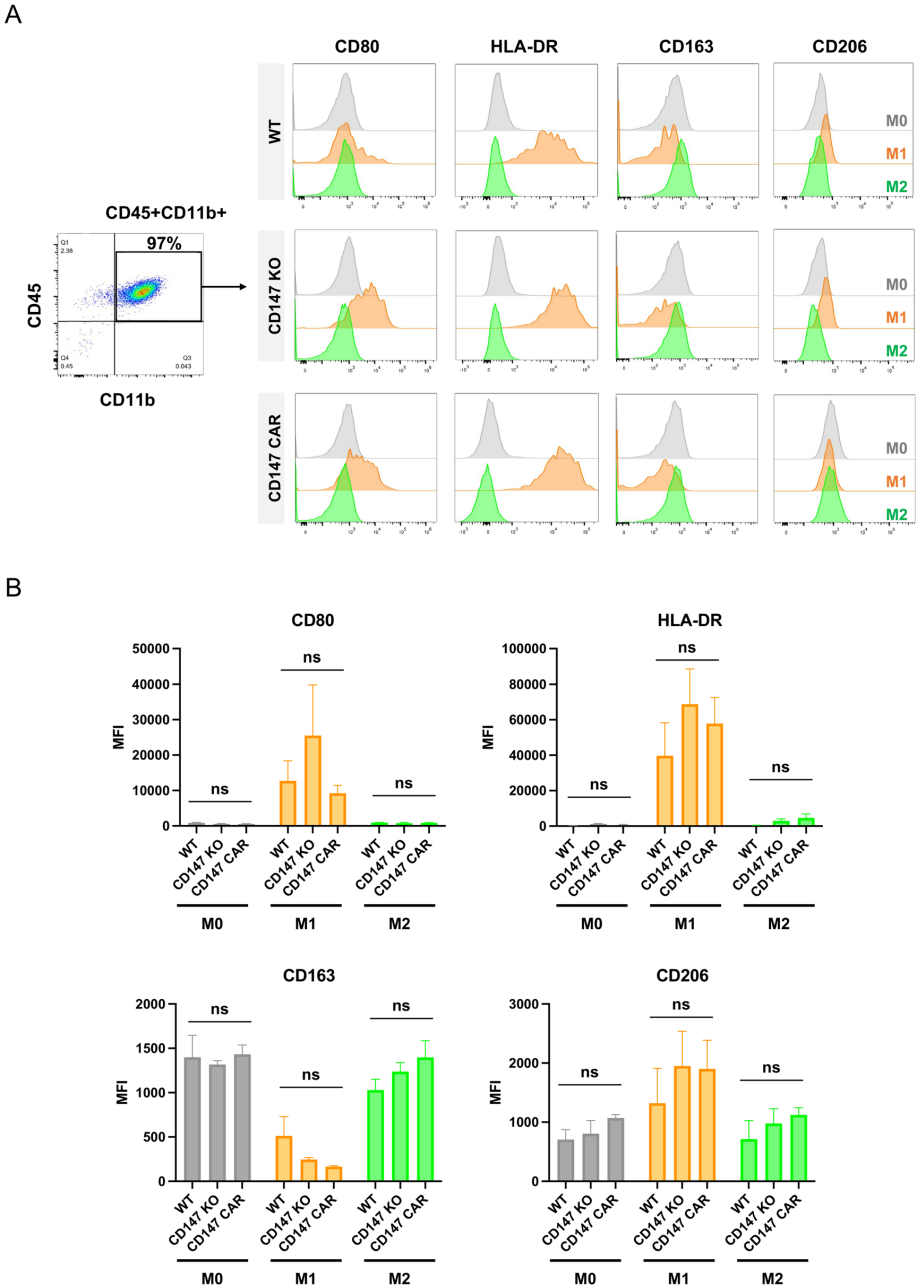
Flow cytometric analysis of THP-1 after polarization toward M1 and M2 subtypes. **A** Representative flow cytometric analysis of the WT, CD147 KO, and CD147 CAR THP-1 cells after activation with PMA (M0, grey), and polarized into M1 (orange) and M2 (green) macrophages. **B** Mean fluorescence intensity (MFI) of CD80, HLA-DR, CD163, and CD206 of THP-1 cells after polarization. Surface markers include M1 markers (CD80 and HLA-DR), and M2 markers (CD163 and CD206). Statistical significance was calculated using multiple unpaired *t* test analyses, ^ns^indicates *P* > 0.05. Data represent the results from three independent experiments

Macrophage polarization is the process by which macrophage phenotypes are altered in response to signals encountered in their microenvironment. The two main phenotypes are classically activated macrophages type 1 (M1), and alternatively activated macrophages type 2 (M2). Upon stimulation with IFN-γ and LPS, we observed high expression of CD80 and HLA-DR (M1 markers), while CD163 and CD206 (M2 markers) were not expressed (Fig. 4A, B). This indicates that IFN-γ and LPS effectively induced the WT, CD147 KO, and CD147 CAR THP-1 macrophages into the M1 subtype. In contrast, CD163 was expressed in both M0 and M2 macrophages, whereas CD206 was not highly expressed in M2 but was slightly expressed in M1 when stimulated with IL-4 and IL-13. However, the expression pattern of CD markers in CD147 KO and CD147 CAR was similar to WT cells. These findings suggest that our engineering strategy to generate the CD147 KO and CD147 CAR THP-1 cells did not interfere with the typical macrophage phenotype.

### The CD147 CAR-M exhibited enhanced phagocytosis upon co-culturing with cancer cell lines

**Fig. 5:**
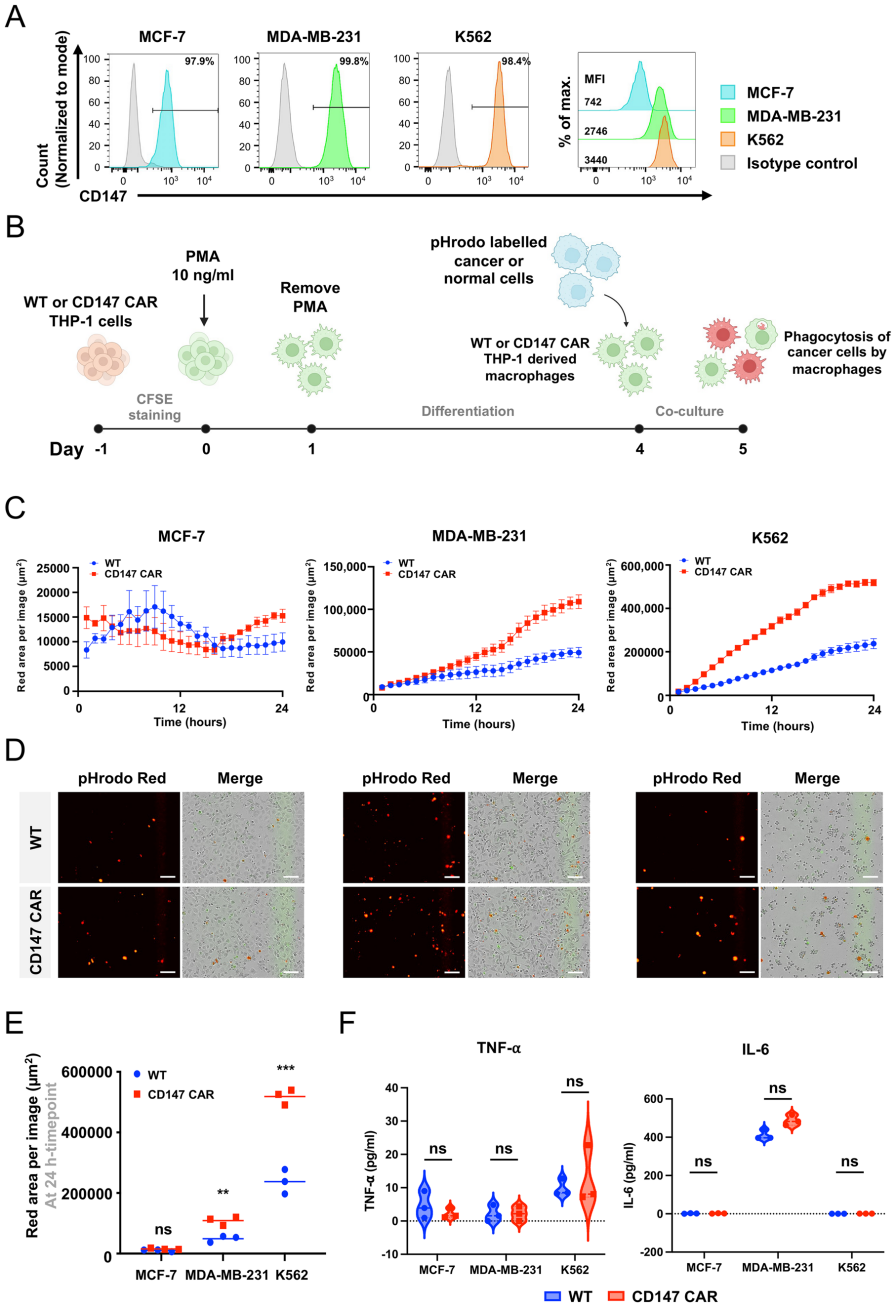
Phagocytosis of cancer cells. **A** Flow cytometric analysis of CD147 expression in cancer cell lines, including MCF-7, MDA-MB-231, and K562, compared to the isotype control. **B** Schematic diagram demonstrating the co-culture experiment of the WT, CD147 KO, and CD147 CAR-M against cancer or normal cells (Created by BioRender.com/Mahidol University). **C** Live-cell time-lapse phagocytosis assay of the WT and CD147 CAR-M against cancer cell lines captured at 1-h intervals for 24 h. The average total red areas per image (µm^2^) are plotted. **D** Representative fluorescent images illustrating the phagocytosis of cancer cells by the WT and CD147 CAR-M in each cancer cell line, scale bar = 100 µm. **E** The comparison of the red area per image (µm^2^) at 24 h of co-culture. **F** TNF-⍺ and IL-6 secretion by the WT and CD147 CAR-M after co-culturing with different cancer cell lines. Statistical significance was calculated using multiple unpaired *t* test analyses, ^ns^indicates *P* > 0.05, **indicates *P* < 0.01, ***indicates *P* < 0.001. Data represent the results from three independent experiments

We next validated the capability of the CD147 CAR-M to target and phagocytose breast cancer cell lines, including MCF-7, MDA-MB-231, and the lymphoblastic cell line, K562. Flow cytometric analysis showed that all cancer cell lines expressed CD147, with different mean fluorescence intensities (MFIs). The MFIs of CD147 in MCF-7, MDA-MB-231, and K562 cells were 742, 2746, and 3440, respectively (Fig. 5A). The WT macrophages and CD147 CAR-M were co-cultured with MCF-7, MDA-MB-231, and K562 cells at the effector to target ratios of 1:3, 1:3, and 1:2, respectively (Fig. 5B). The target cells were stained with pHrodo iFL Red STP ester, which emits a red fluorescent signal inside the phagolysosomes of the THP-1-derived macrophages upon phagocytosis. The results revealed that the CD147 CAR-M exhibited an increased phagocytosis and red fluorescence signals compared to the WT macrophages when co-cultured with K562 and MDA-MB-231 cells over time (Fig. 5C, D). The highest phagocytosis activity of the CD147 CAR-M was observed at 24 h of co-culture with K562 cells, followed by MDA-MB-231 cells. However, there was no significant difference in phagocytosis activity between the CD147 CAR-M and the WT macrophages when co-cultured with MCF-7 cells (Fig. 5E). These results indicate that our CD147 CAR can specifically bind to CD147 on the target cells, leading to phagocytosis. We also examined the CD47 molecule, which serves as a potent ‘don’t eat me’ signal that is often overexpressed in both solid and hematological tumors. The results demonstrated that all three cell lines expressed nearly 100% of CD47. Interestingly, the MFI of CD147 in MCF-7 cells (14836) was higher than those in MDA-MB-231 (12422) and K562 (11308), respectively (Supplementary Fig. 1). Therefore, the phagocytosis activity appeared to be influenced by the levels of both CD147 and CD47 expression, as indicated by the MFI of CD147 and CD47 on the target cells. Nevertheless, analysis of pro-inflammatory cytokine secretion, including TNF-⍺ and IL-6, revealed no significant difference between the WT macrophages and CAR-M across all target cell lines (Fig. 5F).

### Phagocytosis of the CD147 CAR-M against normal cells

**Fig. 6:**
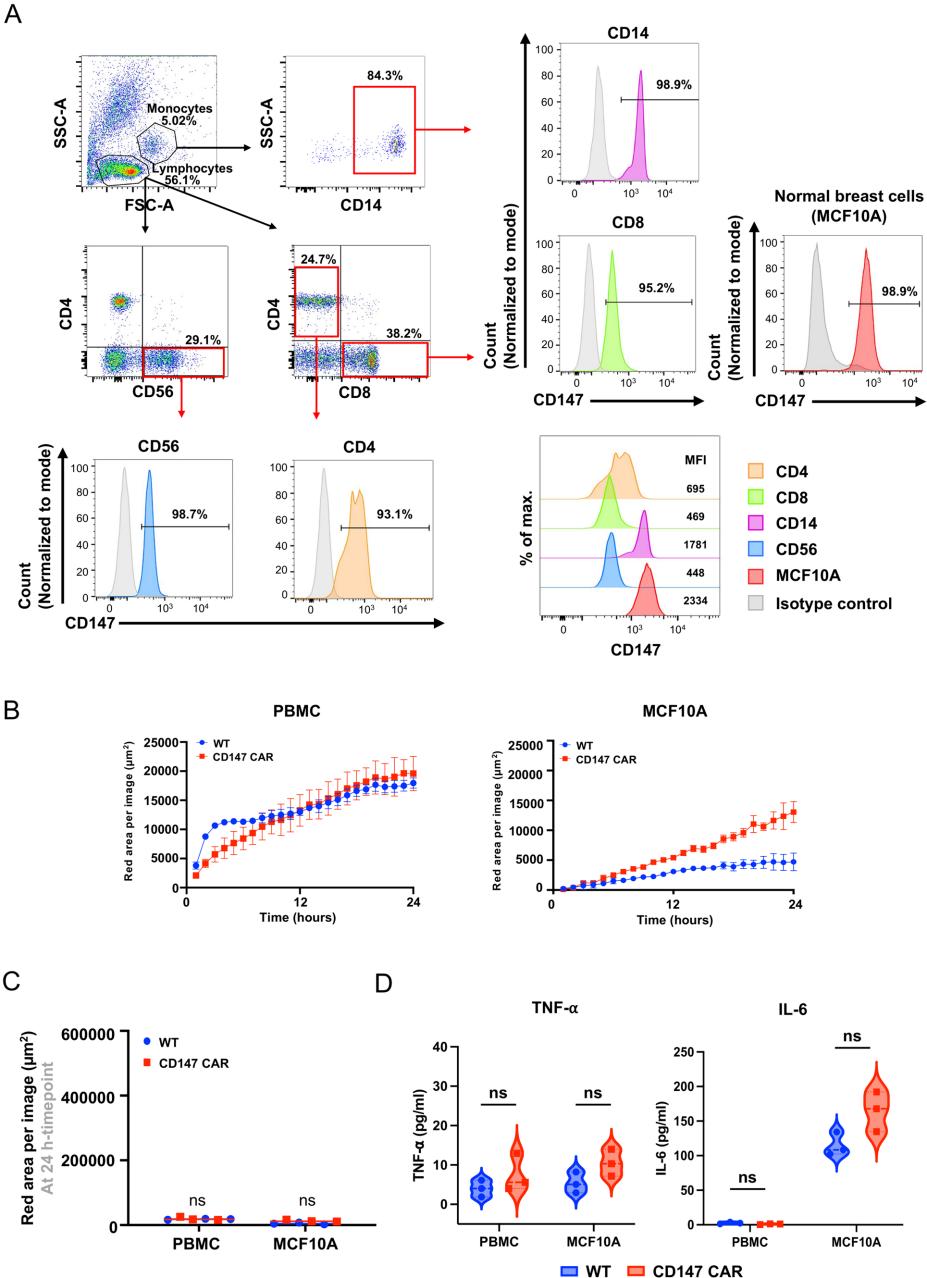
Phagocytosis of normal cells. **A** Flow cytometric analysis of CD147 expression in normal cells, including PBMCs (CD4, CD8, CD56, and CD14 subsets), and MCF10A cell line compared to the isotype control. **B** Live-cell time-lapse imaging of phagocytosis by the WT macrophages and CD147 CAR-M targeting normal cells, recorded at 1-h intervals over 24 h. The graph displays the average total red areas per image (µm^2^) for each condition. **C** The red area per image (µm^2^) at 24 h of co-culture. **D** TNF-⍺ and IL-6 secretion by the WT macrophages and CD147 CAR-M after co-culturing with PBMCs and MCF10A cells. Statistical significance was calculated using multiple unpaired *t* test analysis, ^ns^indicates *P* > 0.05. Data are results from three independent experiments

Another critical aspect of cancer immunotherapy is the risk of on-target, off-tumor toxicities affecting normal cells. To assess the safety of CD147 CAR-M, we performed phagocytosis experiments with normal cells, including MCF10A cells (a normal breast epithelial cell line) and human PBMCs. We first examined the expression of CD147 in each cell type. More than 90% of the PBMCs expressed CD147, with specific subpopulations showing various expression levels: 93.1% of CD4 (MFI = 695), 95.2% of CD8 (MFI = 469), 98.7% of CD56 (MFI = 448), and 98.9% of CD14 (MFI = 1781). Similarly, 98.9% of MCF10A cells expressed CD147 with the MFI = 2334. (Fig. 6A), whereas CD47 expression varied among these cells (Supplementary Fig. 2). We then co-cultured the WT macrophages and CD147 CAR-M with the PBMCs and MCF10A cells at the E:T ratio of 1:3. Even though the results demonstrated an enhanced phagocytosis in both WT and CD147 CAR-M when co-cultured with PBMCs or MCF10A cells over time, no significant differences in the phagocytosis activity between the WT macrophages and CD147 CAR-M cells were observed at 24 h (Fig. 6B, C). Additionally, there was no significant difference in TNF-⍺ and IL-6 secretion (Fig. 6D), suggesting that the CD147 CAR-M did not exhibit off-target cytotoxicity against normal cells despite the presence of CD147 molecules on their surfaces.

## Discussion

Although CAR-T cells have gained FDA approval for hematologic malignancies, their effectiveness against solid tumors remains limited due to challenges such as the immunosuppressive tumor microenvironment (TME) and T cell exhaustion [35]. Macrophages are immune cells abundant in the TME that exhibit anti-tumor activity [36]. Therefore, CAR-M therapies targeting various tumor antigens have been developed [15, 37]. One such target is CD147, a tumor-associated antigen (TAA) upregulated in numerous cancer types, making it an attractive target antigen for cancer immunotherapy [20, 38, 39]. Previous studies utilizing CAR-T and NK targeting CD147 have shown promising results in HCC [29]. However, the potential of CAR-M targeting CD147 has not been investigated.

In this study, we used the THP-1 monocytic cell line as a model to develop CD147 CAR-M and examined the phagocytosis activity against cancer cells. Given that the THP-1 cells naturally overexpress CD147 molecules [40, 41], we initially performed the CRISPR/Cas9-mediated gene knockout to eliminate the CD147 molecule before introducing the CD147 CAR (scFv M6-1B9) using lentiviral vectors. Our findings demonstrated that both CD147 gene knockout and CD147 CAR lentiviral transduction did not interfere with normal cell functions, including phagocytosis of zymosan bioparticles and polarization into M1 and M2 macrophages. We only observed an upregulation of the M2 marker CD163, but not CD206, after polarization with IL-4 and IL-13. This result was consistent with the previous study that showed no change in CD206 expression in the THP-1 cells after polarization with IL-4 and IL-13 [42].

The scFv M6-1B9 has been shown to target a functional epitope within domain 1 of the CD147 molecule [34, 43]. Previous studies have demonstrated that the M6-1B9 intrabody, when bound to the CD147 molecule, inhibits CD147 cell surface expression and reduces the aggressiveness of multiple cancers [31, 44, 45]. Therefore, we employed the scFv M6-1B9 as the extracellular target binding domain for the CD147 CAR. Additionally, the choice of cytoplasmic domain is critical for initiating downstream signaling. Previous studies comparing the CD3ζ intracellular domains, commonly used in CAR T cells with the FcγR signaling domain have demonstrated that both can trigger phagocytosis of cancer cells with similar efficiency in macrophages derived from THP-1 cells [46], HSPC [46] and iPSCs [17, 47]. This is because they both function through the same cytosolic domain, Immunoreceptor Tyrosine-based Activation Motifs (ITAMs) [13]. We selected the human Fc-gamma receptors (FcεRIγ) for the intracellular signaling domain since this receptor is naturally present in macrophages and induces antibody-dependent cellular cytotoxicity and phagocytosis upon binding to its ligand in both cancer and infectious diseases [48, 49]. Our results showed that the CD147 CAR-M with the intracellular signaling domain, human FcεRIγ, could trigger phagocytosis when co-cultured with the CD147-expressing cancer cell lines.

As mentioned earlier, CD147 is expressed in both normal and cancer cells, highlighting the importance of determining the on-target off-tumor toxicity of CD147 CAR-M in normal cells. In this study, we selected three different types of cancers (K562, MCF-7 and MDA-MB-231), and two normal cell types. Our results confirmed the specificity of CD147 CAR, demonstrating its selective binding to CD147 on cancer cells and induction of phagocytosis. Similarly, the previous study showed that the CD147 CAR-T cells exhibited no cytotoxicity against normal T cells, CD147 KO Jurkat cells, or normal stomach fibroblasts [50]. Moreover, administration of the CD147 CAR-NK cells into non-tumor-bearing humanized NSG mice resulted in a slight increase in body weight, suggesting no severe on-target/off-tumor toxicity in vivo [29].

Recently, multiple CAR-M approaches have emerged as alternative treatments for cancer. CAR-M targeting HER2-overepressing tumors is currently in phase I clinical trial (NCT04660929), offering valuable insights into the efficacy and safety of future CAR-M therapies. However, there are several critical concerns regarding macrophage-based cell therapy, such as the polarization patterns within the TME. Tissue-associated macrophages (TAMs) in the TME consist of M1 and M2 macrophages [51]. It is evident that cancer cells within the TME directly regulate the polarization of TAMs. As tumors progress, the M2-like population increases, promoting tumor growth, invasion, and metastasis [52, 53]. As a result, there has been an attempt to suppress the M2-like phenotype by incorporating the toll-like receptor 4 intracellular toll/IL-1R (TIR) in the signaling domain of CAR in the iPSC-derived macrophages (CAR-iMACs) to maintain the M1-like macrophage phenotype. This enhancement resulted in improved anti-tumor activity and a strong M1 polarization phenotype in the in vitro and in vivo studies [47]. Furthermore, challenges exist in obtaining primary monocytes from cancer patients, especially after chemotherapy, and in genetically engineering primary macrophages [13, 17]. Given their unlimited proliferation, differentiation potential, and ease of genetic modification, iPSCs could be an excellent source for generating a large number of engineered CAR-iMACs, as evidenced in the previous studies targeting CD19 [17], and Glypican-3 (GPC3)-expressing cells [47]. Lastly, the use of murine scFvs in CAR construct may lead unexpected immune response in patients, which potentially limiting CAR-M persistence [54]. Therefore, it is necessary to develop humanized scFv to reduce potential immunogenicity when applied to patients [55].

Taken together, our data demonstrate that the CD147 CAR-M exhibits typical macrophage characteristics, including engulfment of zymosan bioparticles and differentiating into M1 and M2 macrophages. Moreover, the CD147 CAR-M effectively phagocytose cancer cell lines while showing minimal phagocytosis of normal cells. However, it is noteworthy that future functional experiments should include a group of THP-1 cells transduced with an irrelevant CAR, such as one based on FMC63, to serve as a control CAR, and CD147-deficient cells as target cells to determine off-target effects. Given the promising potential of CAR-iMACs as an alternative cancer treatment, future research will focus on evaluating the efficiency of CD147 CAR in the iPSC platform to provide an unlimited cell source and off-the-shelf cell products. Additionally, combining CAR-M with CAR-T or CAR-NK therapies may further enhance the efficacy of cancer immunotherapy for solid cancers.

### Supplementary Information

Below is the link to the electronic supplementary material.

Supplementary file1 (DOCX 567 KB)

## Data Availability

No datasets were generated or analysed during the current study.
