# Supplementary material for: Engineered CD147-CAR macrophages for enhanced phagocytosis of cancers
Source: Cancer Immunol Immunother. 2024 Jul 2;73(9):170. doi: 10.1007/s00262-024-03759-6 (PMC11219683; doi:10.1007/s00262-024-03759-6)
Supplement: Supplementary file 1 — Supplementary file1 (DOCX 567 KB) [file 262_2024_3759_MOESM1_ESM.docx]

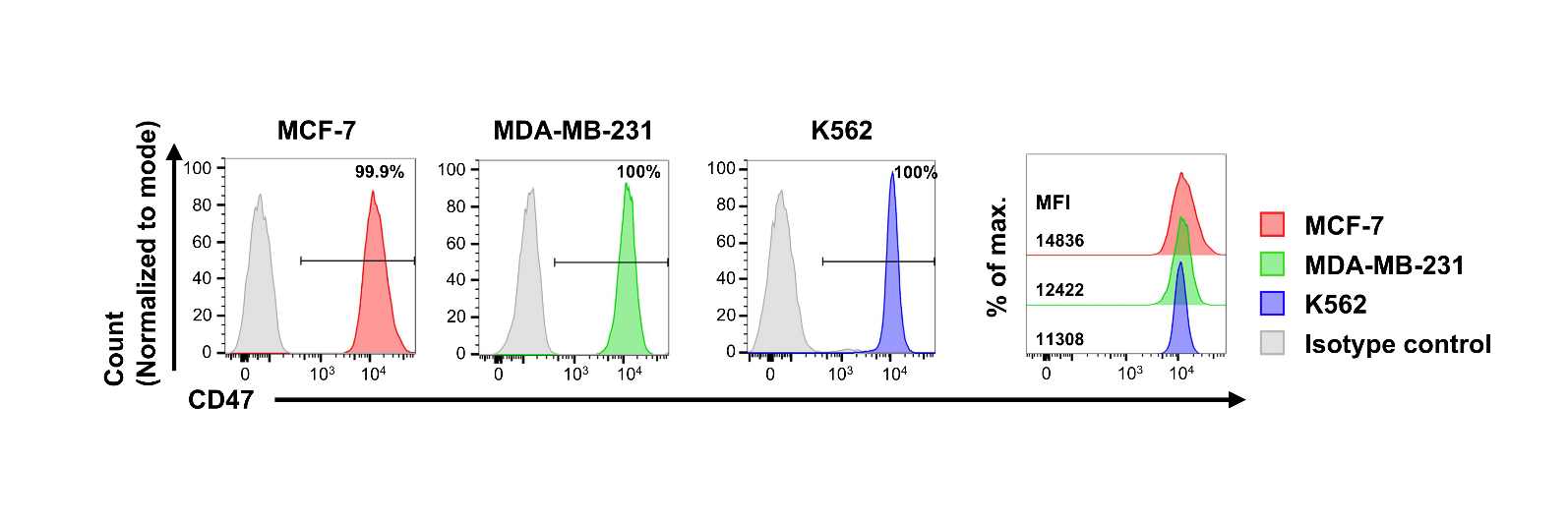


**Supplementary figure 1. CD47 expression on cancer cell lines.** Flow cytometric analysis of CD47 expression in cancer cell lines, including MCF-7, MDA-MB-231, and K562, compared to the isotype control.


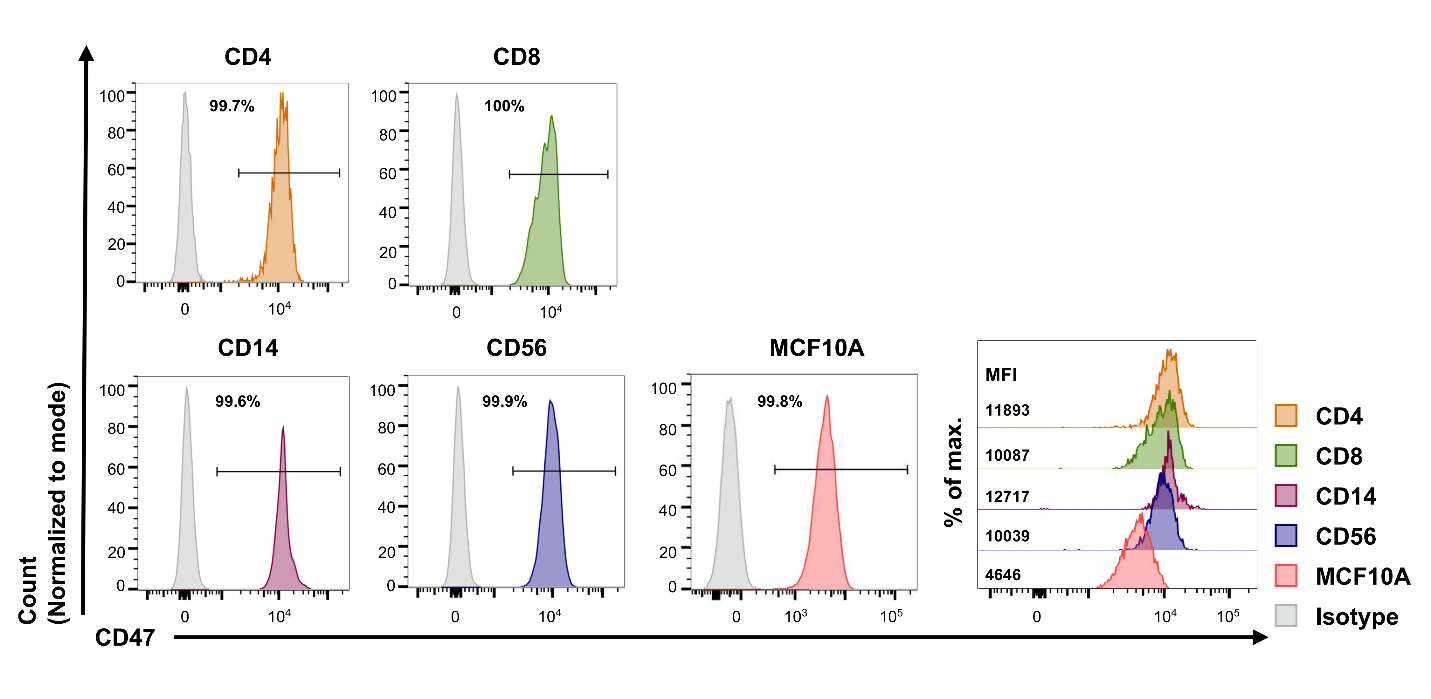


**Supplementary figure 2. CD47 expression on normal cells.** Flow cytometric analysis of CD47 expression in normal cells, including PBMCs (CD4, CD8, CD56, and CD14 subsets), and the MCF10A cell line compared to the isotype control.
